# Gamma-Ray-Induced Structural Transformation of GQDs towards the Improvement of Their Optical Properties, Monitoring of Selected Toxic Compounds, and Photo-Induced Effects on Bacterial Strains

**DOI:** 10.3390/nano12152714

**Published:** 2022-08-07

**Authors:** Sladjana Dorontic, Aurelio Bonasera, Michelangelo Scopelliti, Olivera Markovic, Danica Bajuk Bogdanović, Gabriele Ciasca, Sabrina Romanò, Ivica Dimkić, Milica Budimir, Dragana Marinković, Svetlana Jovanovic

**Affiliations:** 1 Vinča Institute of Nuclear Sciences—National Institute of the Republic of Serbia, University of Belgrade, P.O. Box 522, 11000 Belgrade, Serbia; 2 Department of Physics and Chemistry—Emilio Segrè, University of Palermo, 90128 Palermo, Italy; 3 Interuniversitario Nazionale per la Scienza e Tecnologia dei Materiali (INSTM), Palermo Research Unit, Viale delle Scienze, Bld. 17, 90128 Palermo, Italy; 4 Department of Chemistry, Institute of Chemistry, Technology and Metallurgy, University of Belgrade, Njegoševa 12, 11000 Belgrade, Serbia; 5 Faculty of Physical Chemistry, University of Belgrade, P.O. Box 47, 11158 Belgrade, Serbia; 6 Dipartimento di Neuroscienze, Sezione di Fisica, Università Cattolica del Sacro Cuore, 00168 Rome, Italy; 7 Fondazione Policlinico Universitario Agostino Gemelli IRCCS, 11158 Rome, Italy; 8 Faculty of Biology, University of Belgrade, Studentski Trg 16, 11158 Belgrade, Serbia

**Keywords:** graphene quantum dots, N-doping, gamma-irradiation, photoluminescence, carbofuran, 3-amino-1,2,4-triazole, detection, antibacterial effects

## Abstract

Structural modification of different carbon-based nanomaterials is often necessary to improve their morphology and optical properties, particularly the incorporation of N-atoms in graphene quantum dots (GQDs). Here, a clean, simple, one-step, and eco-friendly method for N-doping of GQDs using gamma irradiation is reported. GQDs were irradiated in the presence of the different ethylenediamine (EDA) amounts (1 g, 5 g, and 10 g) and the highest % of N was detected in the presence of 10 g. N-doped GQDs emitted strong, blue photoluminescence (PL). Photoluminescence quantum yield was increased from 1.45, as obtained for non-irradiated dots, to 7.24% for those irradiated in the presence of 1 g of EDA. Modified GQDs were investigated as a PL probe for the detection of insecticide Carbofuran (2,2-Dimethyl-2,3-dihydro-1-benzofuran-7-yl methylcarbamate) and herbicide Amitrole (3-amino-1,2,4-triazole). The limit of detection was 5.4 μmol L^−1^ for Carbofuran. For the first time, Amitrole was detected by GQDs in a turn-off/turn-on mechanism using Pd(II) ions as a quenching agent. First, Pd(II) ions were quenched (turn-off) PL of GQDs, while after Amitrole addition, PL was recovered linearly with Amitrole concentration (turn-on). LOD was 2.03 μmol L^−1^. These results suggest that modified GQDs can be used as an efficient new material for Carbofuran and Amitrole detection. Furthermore, the phototoxicity of dots was investigated on both Gram-positive and Gram-negative bacterial strains. When bacterial cells were exposed to different GQD concentrations and illuminated with light of 470 nm wavelength, the toxic effects were not observed.

## 1. Introduction

Since they were first reported in 2008 by Ponomarenko and Geim [1], graphene quantum dots (GQDs) have been attracting attention in different scientific fields of research [2]. GQDs are 0D fragments of graphene sheets, with a diameter of less than 100 nm and thickness below 10 graphene layers [3,4]. GQDs are built from graphene core and oxygen functional groups (hydroxyl, carboxyl, epoxy, and carbonyl) bound at their surface and the edges [5]. Graphene core consists of sp^2^-hybridized carbon atoms organized in a honeycomb network. The functionalized surface is responsible for their solubility in water, and polar organic solvents, at a very high concentration such as 10 mg mL^−1^ [6]. The unique physicochemical, electronic, and optical properties such as chemical stability, dispersibility [6,7], tunable band-gap (~1.5 eV) [8], stable photoluminescence (PL) in the visible part of the spectrum, absorption in the UV region [9], resistance to photobleaching [10], as well as non-toxicity [11] and biocompatibility [12], leads to their investigation for sensing [13], bioimaging [12], photocatalysis [14], and energy storage applications [15].

The electronic and optical properties of GQDs can be improved by various approaches [16]. Heteroatom doping is an effective method to easily tune the band-gap of GQDs, and improve their PL quantum yield (QY) [16]. Among the dopants, nitrogen is recognized as a promising candidate for heteroatom doping of GQDs due to its similar atomic radius (0.70 Å) compared to carbon (0.77 Å) as well as similar electronegativity (X_N_ = 3.04 and X_C_ = 2.55) [17]. Incorporated N-atoms modulate excited states of GQDs and increase a radiative transition, leading to improvements in PL properties [7].

In recent years, there has been a growing interest in the application of gamma irradiation as a tool for a simple and eco-friendly structural modification of different carbon-based nanomaterials [18,19]. Li et al. introduced N-atoms in graphene oxide (GO) structure in form of amino groups using ethylenediamine (EDA), a source of N-atoms, at irradiation doses from 5.3 to 35.3 kGy [20]. Later, He et al. irradiated GO at a dose of 200 kGy in the presence of different EDA weight ratios (1:0, 1:5, 1:10, and 1:20) [21]. Milenković et al. fabricated N,S-doped GQDs at irradiation doses from 25 to 200 kGy in the presence of isopropyl alcohol and L-cysteine as a source of heteroatoms [22]. After irradiation, PL QY was increased from 1.45 to 21.60% for GQDs irradiated at a dose of 25 kGy.

GQDs show great potential as PL probes for pesticides [23,24,25]. Traditional commercial methods for pesticide detection are liquid chromatography, high-performance liquid chromatography, gas chromatography, capillary electrophoresis, and atomic absorption [26,27]. These are highly sensitive, but expensive instrumentation, the complicated procedure of sample preparation and the use of toxic chemicals in pretreatments are the main disadvantages [28]. To overcome these issues, researchers are working on the development of new, cost-effective, less toxic, and operationally simple methods for pesticide monitoring [29]. GQDs are examined as PL probes for various pesticides, such as: dichlorvos (limit of detection—LOD 0.778 μmol L^−1^) [30], omethoate (LOD 0.029 pmol L^−1^), ethion (LOD 0.0208 μmol L^−1^) [23], malathion (LOD 0.5 μmol L^−1^), paraquat (LOD 19 μg L^−1^) [31,32,33], glyphosate (LOD 0.05323 nmol L^−1^) [25], diazinon (LOD 0.4 nmol L^−1^) [34], imidacloprid (LOD 0.007 ppm) [24], etc. [13]. The main mechanism in all reported examples is based on luminescence turn-off/turn-on. GQDs PL intensity was quenched (turn-off) by metal ions such as Hg^2+^ [33] or biomolecules such as aptamer [23]. After the addition of pesticides in the GQDs/quencher system, PL of GQDs was recovered (turn-on) due to a higher affinity of the quencher for pesticide molecules than for GQDs [33].

In this paper, we aim to further explore the versatility of GQDs in detection protocols and to lower the current detection limits. Thus, GQDs were fabricated using a top-down electrochemical approach [35]. Water dispersions of GQDs were mixed with different amounts of EDA (1 g, 5 g, and 10 g), and gamma-irradiated at a dose of 200 kGy. The applied dose was chosen according to a previous study where the nitrogen atoms were incorporated at 200 kGy in the highest percent (3.16%) [35]. After treatment with gamma-rays, the morphology of obtained N-doped GQDs was examined using atomic force microscopy (AFM) and dynamic light scattering (DLS). The surface charge was investigated by measuring ξ-potential. The structure of pristine and irradiated GQDs was analyzed using Fourier-transformation infrared (FTIR), X-ray photoelectron (XPS), and nuclear magnetic resonance spectroscopies (NMR). Optical properties were studied by PL and UV-Vis spectroscopies. Irradiated N-doped GQDs were tested as a PL probe for direct detection of carbamate insecticide carbofuran (CF) [13], and for turn-off/turn-on detection of azaheterocyclic compound 3-amino-1,2,4-triazole using Pd(II) ions as a PL quencher.

## 2. Materials and Methods

### 2.1. Chemicals and Materials

Graphite electrodes (99.999% purity, Ø = 3.05 mm) were purchased from Ringsdorff-Werke GmbH (Bonn, Germany). Ethanol (96 vol%), sodium hydroxide, and acetone were purchased from Fisher Scientific (Loughborough, Leicestershire, UK). Dialysis bags (MCWO 3.5 kDa) were bought from Spectrum Laboratory Inc. (San Pedro St., Gardena, CA, USA). Ethylenediamine (EDA) (≥99.5%) was purchased from Carl Roth GmbH (Karlsruhe, Germany), while Palladium (II) chloride (PdCl_2_) was purchased from Thermo Fisher GmbH (Kandel, Germany). Carbofuran and 3-amino-1,2,4-triazole were purchased from Merck/Sigma-Aldrich (Darmstadt, Germany).

### 2.2. Synthesis of Graphene Quantum Dots

GQDs were synthesized by the previously described electrochemical approach using graphite electrodes as a starting material [35]. This sample was labeled as p-GQDs. N-doped GQDs were prepared by gamma irradiation of p-GQDs at a dose of 200 kGy in the presence of three different amounts of EDA (1 g, 5 g, and 10 g). For gamma irradiation, water dispersion of p-GQDs in the concentration of 1 mg mL^−1^ was sonicated for 30 min. Different amounts of EDA were added and samples were purged with Ar. These mixtures were irradiated at a dose of 200 kGy. As a radioactive source, a ^60^Co gamma source was used in the gamma sterilization facility at the Vinča Institute. After irradiation, samples were dialyzed. Samples of GQDs irradiated at 200 kGy with different concentrations of EDA were labeled as GQD-EDA/1 g, GQD-EDA/5 g, and GQD-EDA/10 g.

### 2.3. Characterization of Graphene Quantum Dots

#### 2.3.1. Atomic Force Microscopy (AFM)

The morphology of GQDs was analyzed using AFM. The concentration of all GQDs dispersions used for deposition was 0.28 mg mL^−1^. Samples were prepared by spin-coating deposition (3500 rpm, 1 min) on mica and recorded using Quesant AFM (Agoura Hills, CA, USA) which was operating in the tapping mode, in the air, at room temperature. Standard silicon tip (NanoAndMore Gmbh, Wetzlar, Germany) was used with the constant force of 40 N m^−1^. AFM tips that are used in these measurements had the following characteristics: radius minor than 10 nm, 17 μm height, set back of 15 μm, the half cone angle of 20° ÷ 25° along the cantilever axis, 25° ÷ 30° from the side, and 10° at the apex. AFM images were analyzed using Gwyddion 2.58 software [36]. Due to the small diameter of GQDs, the accuracy of their lateral size was increased by applying the tip deconvolution. The real GQDs diameters were estimated using the following equation:

r_c_ = r (cos θ_0_ + (cos^2^ θ_0_ + (1 + sin θ_0_)(−1 + (tan θ_0_/cos θ_0_) + tan^2^ θ_0_))^1/2^)

r_c_ is the AFM radius of a particle, r is the particle radius, and θ_0_ is the mean half angle of the tip [37]. The histograms of diameter distribution were calculated using 3 different AFM images with the size of 25 × 25 μm^2^ for each GQDs sample.

#### 2.3.2. Dynamic Light Scattering and ξ Potential Measurement

DLS and ζ-potential measurements were acquired at an angle of 173° with a Zetasizer Nano ZS instrument (Malvern, Herrenberg, Germany) equipped with a 633 nm He-Ne laser. Measurements were performed at a fixed position (4.65 mm) with an automatic attenuator and controlled temperature (20 °C). Before measurements, GQD dispersions (0.2 mg/mL) were sonicated with a tip sonicator for 30 min, in ice. For each sample, five independent measurements were averaged, and the number weighted size distribution of the particles was retrieved. Solvent-resistant micro cuvettes(ZEN0040) have been used for experiments with a sample volume of 40 μL. DLS measures the intensity based on the autocorrelation function:

G2(τ) = 〈I(t + τ)I(t)〉/〈I〉(1)

where τ is the lag time and the brackets represent the ensemble average. The G2(τ) can be related to the field autocorrelation function g_1_(t) through the Siegert relation

G2(τ) = 1 + β|g1(t)|^2^ (2)

where β is an instrumental constant equal to 1 in our setup. ζ-potential of GQDs was calculated from the electrophoretic mobility using the Henry correction to Smoluchowski’s equation, implemented in the Z-sizer data-analysis software (Zetaseizer Software 7.13, Malvern Panalytical, 2002 (Worcestershire, UK). For this purpose, 5 independent measurements at pH = 7 were averaged.

#### 2.3.3. Infrared Spectroscopy with Fourier Transformation (FTIR)

For FTIR analysis, powdered samples of GQD-EDA/1 g, GQD-EDA/5 g, and GQD-EDA/10 g, were mixed with KBr and compressed into pellets. Spectra were recorded using an FTIR spectrometer (Thermo Nicolet iS20, Waltham, Massachusetts, MA, USA) in the range of 4000–400 cm^−1^ at 32 scans per spectrum at 4 cm^−1^ resolution. For spectra analysis we used OMNIC 9 software version 9.9.509 by Thermo Fisher Scientific Inc. (Waltham, Massachusetts, MA, USA).

#### 2.3.4. X-ray Photoelectron Spectroscopy (XPS)

XPS spectra of GQD-EDA/1 g, GQD-EDA/5 g, and GQD-EDA/10 g were acquired using a PHI5000 VersaProbe II scanning microprobe (ULVAC-PHI, Inc.; Chigasaki, Japan), operating with a monochromatic Al K_α_ source (1486.6 eV). Used X-ray beam had Ø 100 μm (25 W, 15 kV); low-resolution surveys were collected using pass energy (PE) of 117.000 eV, with a resolution of 1.0 eV; high-resolution scans were collected with a PE of 23.500 eV, with a resolution of 0.05 eV. Collected electrons were analyzed in FAT mode. The samples were prepared by drop-casting deposition over aluminum foil; during the data acquisition, the sample surface was kept at an angle of 45° with respect to the analyzer; charge neutralization was used during all the experiments by means of both electrons and positive ions (Ar⁺).

#### 2.3.5. Nuclear Magnetic Resonance Spectroscopy (NMR)

For ^1^H NMR measurement, samples of GQDs-EDA/1 g, GQDs-EDA/5 g, and GQDs-EDA/10 g were prepared by dissolving in D_2_O:CF_3_COOD 10:1 mixture. Spectra were recorded on a Bruker Avance II 300 MHz spectrometer (Billerica, Massachusetts, MA, USA); 128 scans were performed for the acquisition of each spectrum.

#### 2.3.6. UV-Vis Spectroscopy

Absorption spectra of GQD-EDA/1 g, GQD-EDA/5 g, and GQD-EDA/10 g samples were recorded using LLG-uniSPEC 2 Spectrophotometer (Lab Logistic Group, Meckenheim, Germany) in the wavelength range of 200–800 nm, at room temperature. The concentration of GQDs was 0.25 mg mL^−1^. All spectra were recorded in ultrapure water.

#### 2.3.7. Photoluminescence (PL) Spectroscopy

To examine photoluminescent properties, samples were dispersed in the concentration of 0.075 mg mL^−1^ in ultrapure water. PL spectra were recorded using the HORIBA Jobin Yvon’s Fluoromax-4 spectrometer (Horiba, Kyoto, Japan) at different excitation wavelengths (300, 320, 340, 360, 380, and 400 nm) in the range of 320–580 nm. Excitation and emission slits were 8 and 2 nm, respectively. Integration time was 0.5 s. All spectra were recorded at room temperature and atmospheric pressure. Relative photoluminescence quantum yields (PL QY) were determined by integrating the area under the fluorescence curves using Equation (3):

QY_GQDs_ = QY_REF_(A_REF_/A_GQDs_)(F_GQDs_/F_REF_)(n_GQDs_/n_REF_)^2^ (3)

where QY is photoluminescence quantum yield, F is the integrated area under the PL curve, A is the absorbance value, n is the refractive index of the solvent, while REF and GQDs are referred to as the used reference (Rhodamine B, PL QY is 31%) and GQDs samples.

#### 2.3.8. Photo-Induced Antibacterial Activity

The antimicrobial activity of GQD-EDA samples has been evaluated against reference Gram-positive strain *Staphylococcus aureus* ATCC 29213, and Gram-negative reference strain *Escherichia coli* ATCC 35218. Bacterial suspensions were prepared in phosphate buffer (1 × PBS, phosphate saline buffer) to a final concentration of 108 CFU mL^−1^.

The microdilution method was used to determine the minimum inhibitory concentration (MIC) and the minimum bactericidal concentration (MBC) of the selected nanoparticles [38]. Two-fold serial dilution of the samples with Luria–Bertani (LB) medium was performed, and each well, except for the sterility control, was inoculated with 20 µL of bacterial suspensions (1 × 108 CFU/mL), reaching a final volume of 200 µL. GQD-EDA samples were tested in the concentration from 0.8–0.025 mg mL^−1^. Besides a negative control, a sterility control was prepared. The 96-well microtiter plates were incubated for 16 h at 37 °C under blue light (470 nm, 15 W) at a distance of 20 cm from the light source. A light irradiance in the proximity of the sample was 19 mW cm^−2^. After incubation, optical density (OD) was read on a spectrophotometer (Epoch microplate spectrophotometer, Agilent, Santa Clara, CA, USA). 22 µL of resazurin was added and the microtiter plates were incubated for an additional 2 h at 37 °C. According to the resazurin reaction, the lowest concentration without color change was defined as MIC. The lowest concentration that did not show bacterial growth after sub-culturing and incubation was defined as MBC.

### 2.4. Detection of Carbofuran and 3-Amino-1,2,4-triazole

#### 2.4.1. Sample Preparation

For CF detection, a series of concentrations from 0 to 100 μmol L^−1^ was made and mixed with water dispersion of GQDs-EDA/1 g. Samples were incubated for 24 h, to establish interactions between CF and GQDs. For 3-amino-1,2,4-triazole detection, water dispersion of GQDs-EDA/1 g was firstly mixed with 15 μmol L^−1^ of Pd^2+^ ions. These mixtures were incubated for 2 min. After that, 3-amino-1,2,4-triazole in the concentration range of 0–150 μmol L^−1^ was added and incubated for 5 min.

#### 2.4.2. Photoluminescence Measurement

PL spectra of GQDs-EDA/1 g in the presence of CF and 3-amino-1,2,4-triazole were obtained using HORIBA Jobin Yvon’s Fluoromax-4 spectrometer at room temperature at 360 nm excitation wavelength. Excitation and emission slits were 8 and 2 nm. Integration time was 0.5 s.

## 3. Results and Discussion

### 3.1. Characterization of GQDs

#### 3.1.1. Morphology Analysis of Gamma-Irradiated GQDs

**Figure 1:**
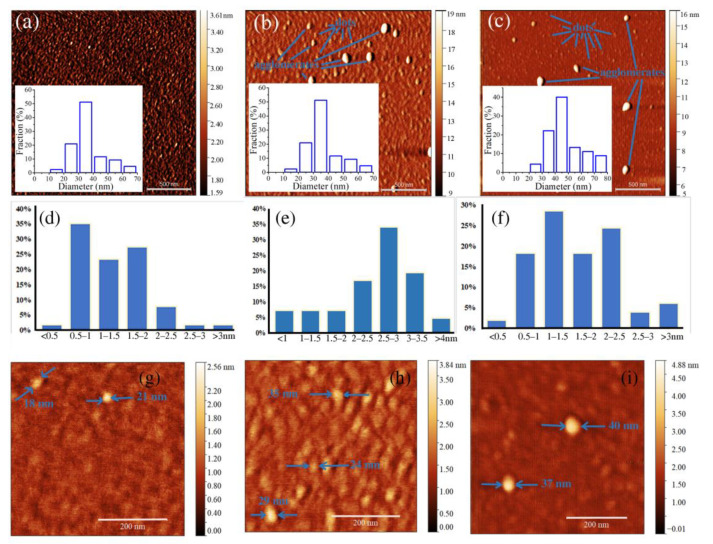
AFM images of GQD-EDA/1 g (**a**), GD-EDA/5 g (**b**), and GQD-EDA/10 g (**c**), with height distributions (**d**–**f**) and large-scale AFM images of isolated dots (**g**–**i**), respectively.

In Figure 1, AFM images with height histograms of GQD-EDA/1 g (a), GQD-EDA/5 g (b), and GQD-EDA/10 g (c) are presented. All images show spherical, well-dispersed particles (indicated with arrows as dots) with few agglomerates presented in the samples GQDs-EDA/5 g and GQDs-EDA/10 g that are marked with blue arrows, in Figure 1b,c. Height distribution diagrams suggest that the largest number of particles in samples GQDs-EDA/1 g, GQDs-EDA/5 g, GQDs-EDA/10 g are 0.5–1, 2.5–3, and 1–1.5 nm high, respectively. The thickness of the individual GQDs indicates its lamellar structure has consisted of 3–5 graphene layers. Average heights of GQD-EDA/1 g, GQD-EDA/5 g, and GQD-EDA/10 g were 1.25 ± 0.13 nm, 2.57 ± 0.39 nm, and 1.58 ± 0.45 nm, respectively. The histograms of diameter distributions are presented in the lower left corner of each large-scale AFM image (a–c), while the high-magnification images are presented in (g–i). The largest fraction of GQDs in samples GQD-EDA/1 g and GQD-EDA/5 g are those with a diameter of around 35 nm, while in the sample GQD-EDA/10 g, the largest fractions are those with a diameter of around 45 nm. The average values were calculated to be 35.5 ± 3.6, 39.3 ± 1.8, and 41.1 ± 3.8 nm for GQD-EDA/1 g, GQD-EDA/5 g, and GQD-EDA/10 g, respectively.

**Figure 2:**
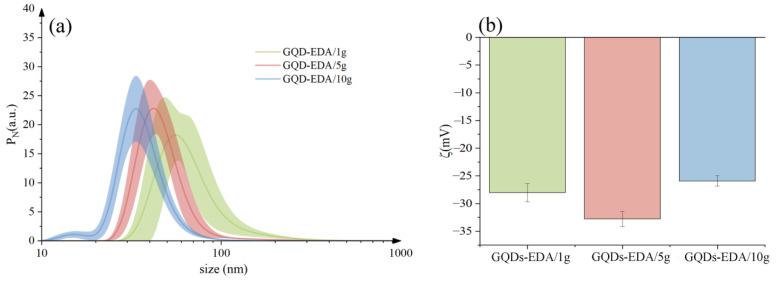
Number-weighted size distributions of the three GQDs compounds measured with DLS (**a**) with the corresponding ζ-potentials (**b**).

The size distribution of GQDs was investigated using DLS and their surface charge was measured by zeta-potential (ζ). The results of DLS and ζ-potential are reported in Figure 2a,b, respectively. All the measured GQDs show a monomodal number-weighted diameter distribution with mode values of 33.4 nm (GQD-EDA/10 g), 42.0 nm (GQDs-EDA/5 g), and 55.7 nm (GQDs-EDA/1 g). The ξ-potential plots are presented in Figure 2b and show that all investigated samples display values in the negative electric region. The following values obtained for GQDs-EDA/1 g, GQDs-EDA/5 g, and GQDs-EDA/10 g were −28.0 ± 1.6 mV, −32.8 ± 1.4, and −25.9 ± 0.9 mV, respectively. An increase of ξ-potential values of irradiated GQDs compared with p-GQDs (−34 eV) [35] was due to a change in the amount of negatively charged functional groups during gamma irradiation in the presence of EDA, probably due to the lower number of carboxyl functional groups.

#### 3.1.2. Structural Characterization of GQDs

**Figure 3:**
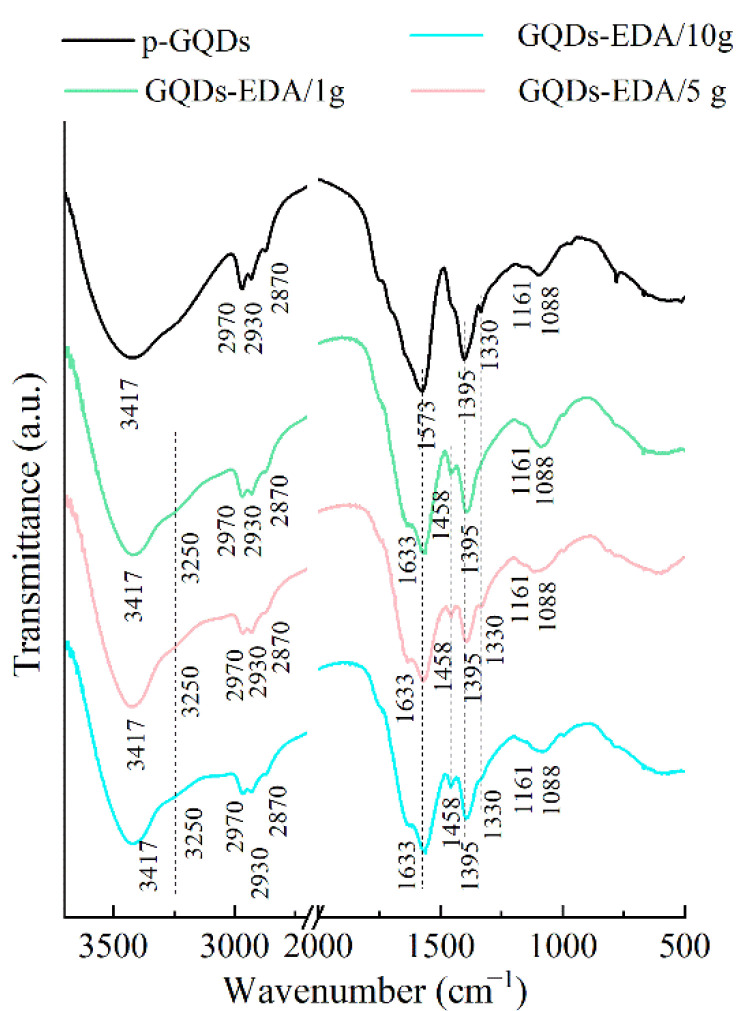
FTIR spectra of p-GQDs, GQDs-EDA/1 g, GQDs-EDA/5 g, GQDs-EDA/10 g.

In Figure 3, FTIR spectra of p-GQDs, GQDs-EDA/1 g, GQDs-EDA/5 g, and GQDs-EDA/10 g are shown. The band at 3417 cm^−1^ located in all spectra stems from the stretching vibrations of O-H bonds in hydroxyl groups attached to carbon atoms. A new band at 3250 cm^−1^ in GQD-EDA/1 g, GQDs-EDA/5 g, and GQDs-EDA/10 g is assigned to N-H bonds in amino groups [35]. The bands at 2970, about 2930, and 2870 cm^−1^ in pristine and irradiated GQDs are attributed to stretching vibrations of C-H bonds in -CH, and -CH_2_ groups [35]. The band located at 1633 cm^−1^ in the spectra of all samples steams from stretching vibrations of C=O bonds in amide functional groups [22]. The high-intensity band at 1572 cm^−1^ detected in the spectrum of the p-GQDs is assigned to stretching vibrations of the C=C bonds in π-conjugated domains of GQDs [35]. In the spectra of irradiated GQDs, this band is slightly shifted at 1567 cm^−1^. This change can be attributed to defect formation in the sp^2^ region of the GQD structure formed due to irradiation. A new band at 1458 cm^−1^ appeared in the spectra of GQDs-EDA/1 g, GQDs-EDA/5 g, and GQDs-EDA/10 g arises from C-N bending in N-C=O functional groups [39]. The bands centered at 1330 cm^−1^ in the spectra of p-GQDs, and GQDs-EDA/5 g_,_ at 1339 cm^−1^ in the spectra of GQDs-EDA/1 g and GQDs-EDA/10 g, as well as the high-intensity band at 1395 cm^−1^ found in all spectra, originate from vibrations of the C-OH bonds [40,41]. The bands at 1161 cm^−1^ observed in all spectra and the one around 1088 cm^−1^ found in the spectra of p-GQDs, GQD-EDA/1 g_,_ and GQDs-EDA/10 g are assigned to asymmetric [42] and symmetric stretching vibrations of the C-O bonds in epoxy groups [22,43].

According to FTIR spectra, all GQDs contain: -OH, -CH_2,_ -CH, -COOH, C=C, and C-O-C functional groups. In N-doped gamma-irradiated GQDs, N-C=O and -NH functional groups were found. These results indicate the incorporation of N atoms in the structure of GQDs using a gamma irradiation dose of 200 kGy.

**Figure 4:**
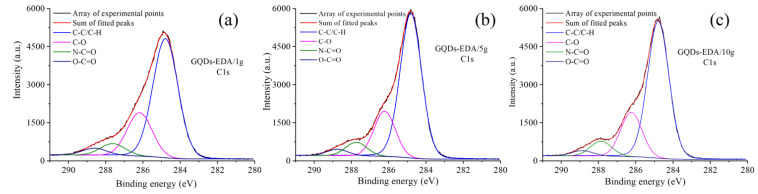
Analysis of high-resolution XPS spectra of C 1s region for samples GQDs-EDA/1 g (**a**), GQDs-EDA/5 g (**b**), and GQDs-EDA/10 g (**c**).

Structural properties of irradiated GQDs also were examined using XPS. The analysis of the C 1s region of XPS spectra showed the presence of C-C/C-H (284.80 eV, used as energy reference for all spectra), C-O (286.18–286.22 eV), N-C=O (287.72–287.74 eV), and O-C=O (288.60–288.87) groups (Figure 4a–c). The relative abundance of each element is shown in Table 1 (in at%).

**Figure 5:**
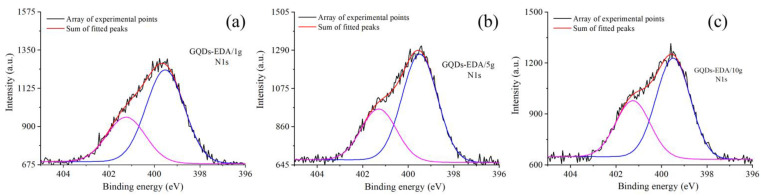
Analysis of high-resolution XPS spectra of N 1s region for samples GQDs-EDA/1 g (**a**), GQDs-EDA/5 g (**b**), and GQDs-EDA/10 g (**c**).

The analysis of the N 1s region showed the occurrence of (at least) two main components, namely at 399.52 eV and 401.24 eV (Figure 5a–c). Both chemical environments are compatible with nitrogen in an organic matrix (organic carbon-bound nitrogen).

Atomic % of C, O, and N in the samples p-GQDs, GQD-EDA/1 g, GQDs-EDA/5 g, and GQDs-EDA/10 g obtained by XPS analysis are presented in Table 1. The first observed change is an increase of carbon content in all irradiated samples, due to the reduction of the part of the oxygen-containing groups during gamma irradiation. Secondly, the oxygen content is significantly decreased in irradiated samples. This change could be attributed to the transformation of O-C=O to N-C=O groups in the irradiation conditions. In line with that, the content of carboxyl groups decreases, while the content of amide groups became higher with the increase in EDA weight. These results are in agreement with ζ-potentials analysis, which showed that gamma irradiation leads to the formation of lees negatively charged particles.

It was calculated that carboxyl groups were 3.76, 2.94, and 2.53% for GQD-EDA/1 g, GQDs-EDA/5 g, and GQDs-EDA/10 g, respectively. The content of amide bonds was similar for all samples, from 6.38 to 6.25, and 7.16%. In the case of C-O, C1s data fitting showed that C-O bonds were 23.54, 20.92, and 21.41% for GQD-EDA/1 g, GQDs-EDA/5 g, and GQDs-EDA/10 g, respectively. The bend that was assigned to C-C/C-H showed that samples contained from 66.32 to 69.90%.

**Figure 6:**
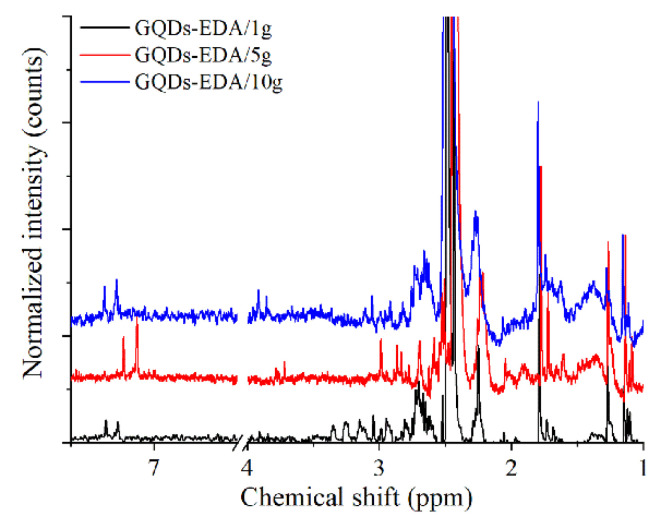
^1^H NMR spectra of GQDs-EDA/1 g (black), GQDs-EDA/5 g (red), and GQDs-EDA/10 g (blue).

Results obtained by ^1^H NMR recording of GQDs-EDA/1 g (Figure 6, black line), GQDs-EDA/5 g (Figure 6, red line), GQDs-EDA/10 g (Figure 6, blue line) show that spectral features are similar for different samples. Signals between 1.00 and 2.50 ppm can be ascribed to the presence of alkyl protons (-CH_3_, -CH_2_-). The sharp, intense signal localized between 2.40–2.45 ppm could be attributed to methyl (or methylene) groups connected to electron-withdrawing moieties. Spectral features between 3.00 and 3.50 ppm could be compatible with -CH_2_- groups bearing an -OH moiety but are less evident in the spectrum recorded for sample GQDs-EDA/10 g. There are low intensity, sharp signals between 6.50–7.50 ppm, which are related to amide groups. However, the presence of amino groups is not excluded, but the typical broad bands at the low field are barely visible due to the limited amount of solubilized material and the weak intensity of the whole spectral profile.

Results obtained by FTIR, XPS, and NMR spectroscopies confirmed that gamma-irradiated GQDs possess C, O, N, and H in their structure. Obtained dots have a larger amount of C in their structure compared to p-GQDs (63% for p-GQDs and around 76% for gamma irradiated), which indicated a chemical reduction of oxygen-containing functional groups. This treatment also induced the incorporation of amino functional groups and the formation of amide bonds.

#### 3.1.3. Optical Properties of GQDs

**Figure 7:**
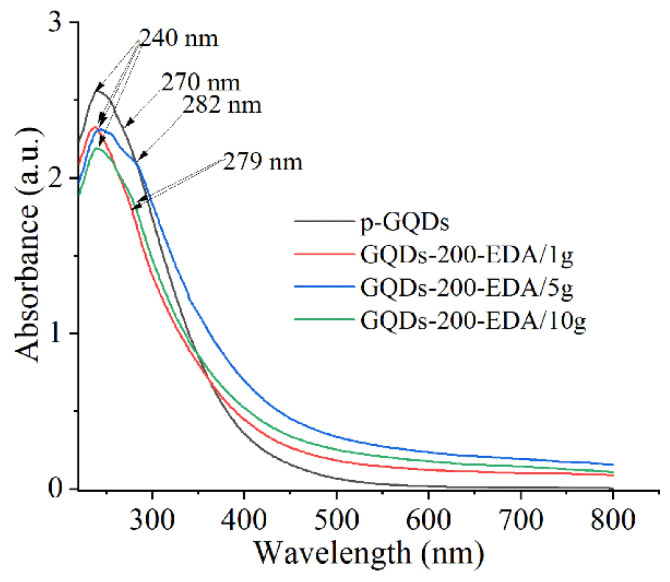
UV-Vis spectra of p-GQDs, GQDs-EDA/1 g, GQDs-EDA/5 g, and GQDs-EDA/10 g.

The optical properties of GQDs were examined using UV-Vis Vis and PL spectroscopies. Absorption spectra of the samples p-GQDs, GQDs-EDA/1 g, GQDs-EDA/5 g, and GQDs-EDA/10 g are presented in Figure 7. For all samples, the high absorption band at about 240 nm is detected. This band is attributed to the π-π* transition of C-C aromatic domains. A weak shoulder band at 270 nm observed in the spectrum of p-GQDs indicates n-π* transitions in C=O bonds. This band is more pronounced and shifted at 279, and 282 nm in the GQD-EDA/1 g, GQDs-EDA/5 g, and GQDs-EDA/10 g, respectively. The shift in this region can be explained by changes in the chemical environment around carbonyl groups in the samples due to functionalization.

**Figure 8:**
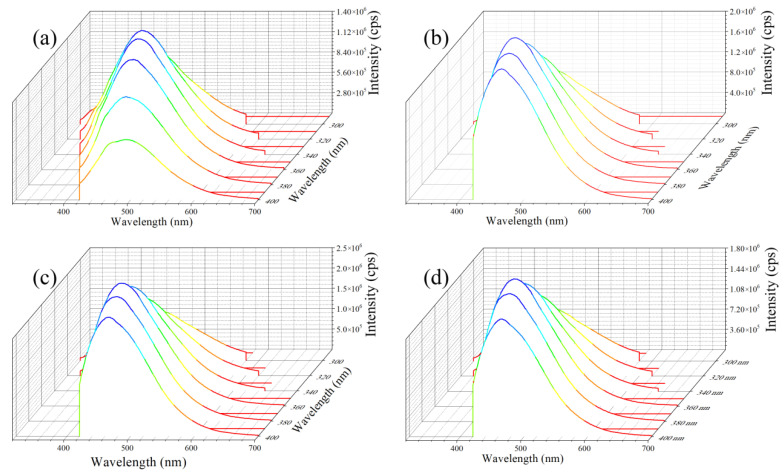
3D PL spectra of p-GQDs (**a**), GQDs-EDA/1 g (**b**), GQDs-EDA/5 g (**c**), and GQDs-EDA/10 g (**d**) recorded at excitation wavelengths in the range of 300–340 nm.

In Figure 8, 3D emission spectra of p-GQDs (a), GQD-EDA/1 g (b), GQD-EDA/5 g (c), and GQD-EDA/10 g (d) were recorded at different excitation wavelength in the range of 300–400 nm are presented. First, p- and irradiated GQDs demonstrate excitation-depended PL, as observed previously [3,44]. This behavior is more pronounced in p-GQDs than in N-doped gamma-irradiated samples. As can be seen in Table 2, the emission peak maximum of p-GQDs is moved from 430 to 492 nm, while in GQD-EDA/1 g, GQD- EDA/5 g, and GQD- EDA/10 g, these shifts were from 441 to 465, from 438 to 465, and from 439 to 466 nm, at the excitation wavelength in the range of 300–400 nm. Excitation-depended PL may result from differently sized dots (according to quantum confinement effect with the increase of particle size, band gap decreases), or different surface chemistry. GQDs surface is rich in trap states such as O-functional groups (–COOH,–OH, and C–O–C), the dangling bonds, and sp^2^/sp^3^ C atoms [45]. Generally, surface trap states are functional groups, oxygen-related disorder-induced localized states, and surface defects [45]. One study suggested that GQD optical emissions stem most probably from continuous defect states (functional groups), and different defect states are the reason for variation in emissions at different wavelengths [46]. Less dependence on the emission peak position from excited light in irradiated GQDs is attributed to the higher uniformity of size and surface state of these particles [47].

**Figure 9:**
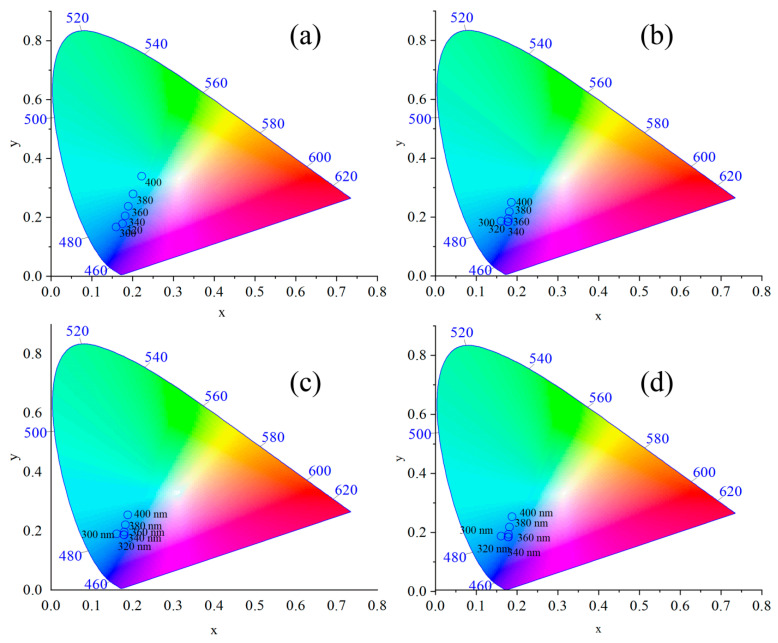
*CIE 1931* chromaticity diagrams of p-GQDs (**a**), GQDs-200-EDA/1 g (**b**), GQDs-EDA/5 g (**c**), and GQDs-EDA/10 g (**d**).

According to the *CIE 1931* chromaticity diagrams, the emission of p-GQDs (Figure 9a) varies from violet-like blue to turquoise part of the spectrum, while all N-doped GQDs exhibit intense blue emission (Figure 9a–c). These diagrams indicate that the position of the emission band center for gamma-irradiated GQDs is less dependent than in the case of p-GQDs. GQDs-EDA/1 g to 10 g still show excitation-dependent PL emission. PL properties of GQDs are controlled by intrinsic and extrinsic factors [48]. It is proposed that the blue emission originated from ordered sp^2^ domains in GQDs (intrinsic state emission) [48]. Additionally, intensive blue emission was reported for N-doped GQDs with amide groups [49]. In this case, control factors for strictly blue emission should be both the surface state and graphene core.

In terms of PL intensity, the highest emission band intensity for p-GQDs was recorded when the excitation wavelength was 340 nm, while GQDs-EDA/1 g at 360 nm, and GQDs-EDA/5 g and GQDs-EDA/10 g, were observed when excited with 380 nm. The next observed change in PL spectra of irradiated samples is an increase of intensity at all excitation wavelengths compared to p-GQDs, whereby maximum value was recorded in the PL spectrum of GQDs-EDA/5 g (Table 2).

In Table 3, the values of PL QY are presented. These data were calculated using Equation (3) and PL spectra recorded at the excitation wavelength of 360 nm. As a reference, Rhodamine B was used. Relative PL QY of p-GQDs, GQDs-EDA/1 g, GQDs-EDA/5 g, and GQDs-EDA/10 g are 1.45, 7.24, 6.77, and 5.82%. Obtained results show that the introduction of N-atoms in GQDs structure led to PL QY enhancement, whereby the highest value was found for GQDs-EDA/1 g.

**Figure 10:**
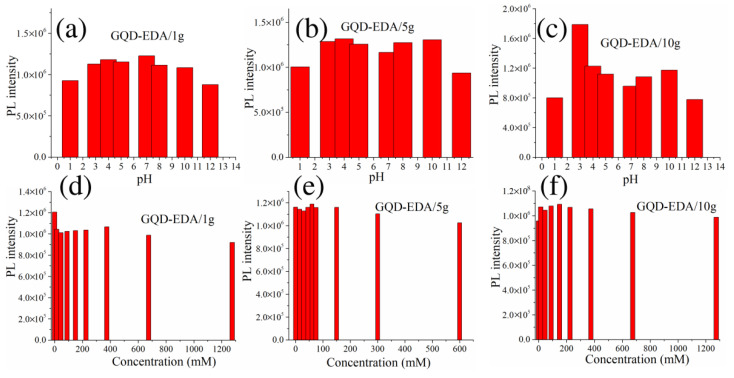
The intensity of the emission bands of the GQDs-EDA/1 g (**a**), GQDs-EDA/5 g (**b**), and GQDs-EDA/10 g (**c**), recorded at λ_exc_ = 360 nm at different pH values and in the presence of the different amount of NaCl, the concentration range 0–1275 mM, (**d**–**f**).

In Figure 10, the effects of pH and ionic strength on the intensity of emission bands are presented. Only very low (such as 1) and very high (12) pH values caused the small lowering in the intensity of emission bands for all three samples. Thus, it can be concluded that PL emission of gamma irradiated GQDs is stable in the pH range 2–10. PL emission was also stable in the presence of different NaCl concentrations, from 15 to 1275 mM.

### 3.2. Detection of CF and 3-Amino-1,2,4-triazole

#### 3.2.1. PL Spectroscopy

**Figure 11:**
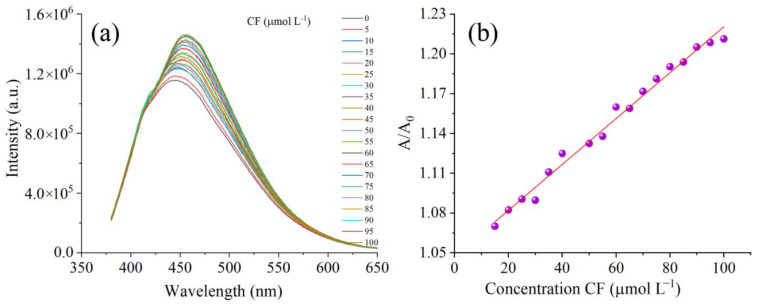
Emission spectra of the GQDs-EDA/1 g recorded at λ_exc_ = 360 nm in the presence of CF in the concentration range 0–100 µmol L^−1^ (**a**), and plot which presents an integrated area under emission peak versus concentration of CF with a linear fitting curve (**b**).

Figure 11a shows PL spectra of GQDs-EDA/1 g in the presence of CF concentration from 0 to 100 μmol L^−1^. It can be seen that PL intensity increases with increasing CF concentration. The linear relationship between PL intensity was determined using equation A/A_0_ = 1 + K_SV_ [Q], where A and A_0_ are integrated areas under the emission peak in the presence and absence of CF, respectively, K_SV_ is Stern–Volmer constant and corresponds to the slope obtained by a linear fit of experimental data presented in Figure 10b, while Q is the concentration of the analyte. Linear relationship is described as: A/A_0_ = 1.04757 + 0.00173 [CF]. A good linear response (R^2^—coefficient of determination = 0.98) was found within a wide concentration range (15–100 μmol L^−1^). LOD was calculated using the equation LOD = 3S_D_/K_sv_, where S_D_ represents the standard deviation of intercept [27]. Obtained LOD was 5.4 μmol L^−1^. In the spectra, a significant shift can be seen of emission peak maximum with increasing in CF concentration (from 442 nm at 0 to 455 nm at 100 μmol L^−1^).

**Figure 12:**
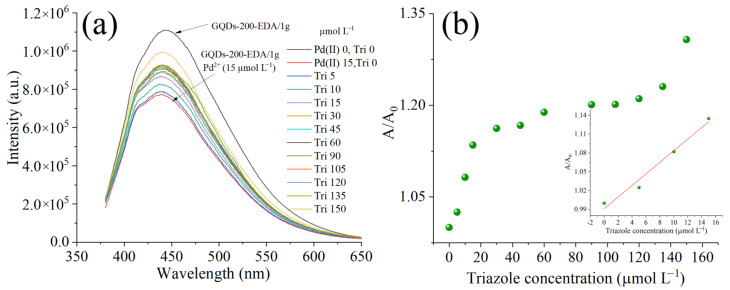
Emission spectra of the GQDs-EDA/1 g, and GQDs-EDA/1 g-Pd^2+^ system recorded at λ_exc_ = 360 nm in the presence of 3-amino-1,2,4-triazole in the concentration range 0–150 µmol L^−1^ (**a**), and plot which presents an integrated area under emission peak versus concentration of 3-amino-1,2,4-triazole (**b**), with a linear fitting curve.

Emission spectra of GQDs-EDA/1 g in the presence of the Pd^2+^ at the concentration of 15 μmol L^−1^ and increasing concentration of 3-amino-1,2,4-triazole from (0–150 μmol L^−1^) are presented in Figure 12a. Obtained results show that Pd^2+^ dramatically quenched a PL intensity of GQDs due to the formation of the GQDs/Pd^2+^ complex. With the increase of 3-amino-1,2,4-triazole concentration in the 3-amino-1,2,4-triazole/GQDs/Pd^2+^ system, PL intensity was recovered. This event is the consequence of the high affinity of 3-amino-1,2,4-triazole molecules toward metal ions [50]. Pd^2+^ ions and 3-amino-1,2,4-triazole build the complex, which leads to the breaking of the bonds between GQDs and Pd^2+^ ions and whereby their PL has been recovered. The plot of the integrated area under emission peak versus concentration of 3-amino-1,2,4-triazole is presented in Figure 12b. Linear relationship between GQDs/Pd^2+^ system and 3-amino-1,2,4-triazole is defined as: A/A_0_ = 0.99123 + 0.00923 [triazole]. The coefficient of determination was 0.97 and this value was observed in the 3-amino-1,2,4-triazole concentration range 0–15 μmol L^−1^. LOD was 3.09 μmol L^−1^.

**Figure 13:**
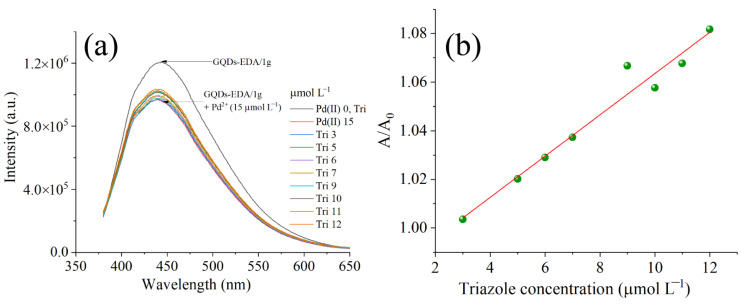
Emission spectra of the GQDs-EDA/1 g and GQDs-EDA/1 g-Pd^2+^ system recorded at λ_exc_ = 360 nm in the presence of 3-amino-1,2,4-triazole in the concentration range 0–12 µmol L^−1^ (**a**), and plot which presents an integrated area under emission peak versus concentration of 3-amino-1,2,4-triazole with a linear fitting curve (**b**).

Based on these results, we examine the detection of 3-amino-1,2,4-triazole in the concentration range of 0–12 μmol L^−1^ (Figure 13a). It can be observed that at these concentrations of pesticide, a PL of GQDs/Pd^2+^ system was recovered linearly in the whole tested concentration range (Figure 13b). Linearity between probe PL intensity and 3-amino-1,2,4-triazole concentration was presented as: A/A_0_ = 0.9787 + 0.00848 [triazole]. In this case, R^2^ and LOD were 0.96 and 2.03 μmol L^−1^, respectively.

Presented results showed that tested PL probe GQDs-EDA/1 g can be applied for direct turn-on detection of CF, and turn-off/turn-on detection of 3-amino-1,2,4-triazole using Pd^2+^ ions as quenching agents.

#### 3.2.2. Detection Mechanism

**Figure 14:**
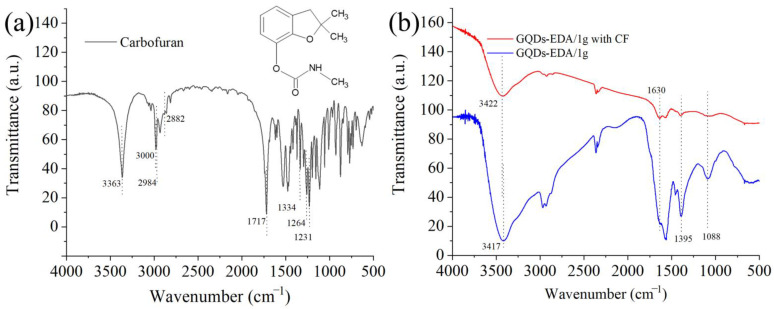
FTIR spectrum and structure of CF (**a**), and FTIR spectra of GQDs-EDA/1 g before and after addition of CF (**b**).

The mechanism of interaction between CF and 3-amino-1,2,4-triazole is resolved using FTIR spectroscopy. In Figure 14a, FTIR spectra of CF, GQDs-EDA/1 g, and GQDs-EDA/1 g-CF are presented. In the FTIR spectrum of CF, bands at 1231 and 1264 cm^−1^ are attributed to stretching vibrations of C-O-C, where one C atom is attached to the aromatic ring, while the other is bonded to the aliphatic structure [51]. A high-intensity band at 1717 cm^−1^ is assigned to the bending vibration of C=O groups [51]. The bands in the range 2882–3000 cm^−1^ correspond to symmetric and asymmetric stretching of -CH_2_ and -CH_3_ moieties [51]. At 1334 and 3363 cm^−1^, bands are assigned to stretching vibrations of C-N, and N-H groups, respectively [51,52].

FTIR spectra of GQDs-EDA/1 g + CF and GQDs-EDA/1 g are compared and presented in Figure 14b. First, the band at 3417 cm^−1^ in the untreated GQDs spectrum from O-H bonds is shifted to 3422 cm^−1^ after CF addition. A small shift in the hydroxyl group band is probably due to hydrogen bond formation between CF and the functional groups at the GQDs surface [52]. A very weak band at around 1630 cm^−1^ observed in the GQDs-EDA/1 g is assigned to the carboxyl functional group. After treatment with CF, this band became defined and more pronounced. That change can be attributed to O–H bending in hydrogen bonds established between CF molecules and carboxyl groups of GQDs [53]. High-intensity bands at 1088 cm^−1^ and 1395 cm^−1^ found in the GQDs-EDA/1 g spectrum from C-O bonds in epoxy groups and C-OH became weaker and they almost disappeared from the spectrum when CF was added to the system. Their lowering may indicate the dissociation of such bonds in the binding mechanism of carbofuran to GQDs [54].

**Scheme 1:**
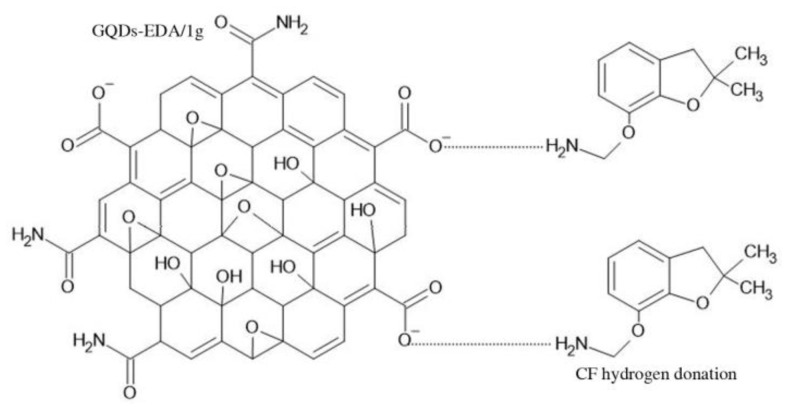
Formation of hydrogen bonds between CF molecules and GQDs carboxyl functional groups at pH 7.

Carbon materials such as sugarcane bagasse biochar [52], and others [53,54,55], adsorb CF from the water via hydrogen bonds. CF at pH > 3.78 acts as an H-acceptor, while -OH groups of biochar play the role of an H-donor. Additionally, it was reported that the N-H group of CF are donated protons and created H-bond with oxygen functional groups of the biochar [52]. According to the FTIR spectrum of the GQDs-EDA/1 g-CF, shifts in the bands assigned to O-H, -COOH, -C-O, and -C-OH groups can be assigned to the formation of hydrogen bonds between CF molecules and O-functional groups of GQDs. Considering a ξ-potential result, carboxyl functional groups on GQDs surface are deprotonated at pH 7, so CF probably served as an H-bond donor in complex with GQDs (Scheme 1).

This binding mechanism is explaining the increase of GQDs PL intensity. In several previous studies, it was found that hydrogen bonds formed between carbon dots and pesticide atrazine [56], B,N,S-doped GQDs and glucosamine [57], and B-doped GQDs and glucose [58] lead to the increase in PL intensity due to structural rigidification. In a rigid structure, intermolecular motions are restricted which caused the blocking of non-radiative channels. Relaxation of excitons was directed through radiative channels increasing GQDs PL intensity. Interaction of GQDs functional groups with CF caused the shift of the emission band.

**Figure 15:**
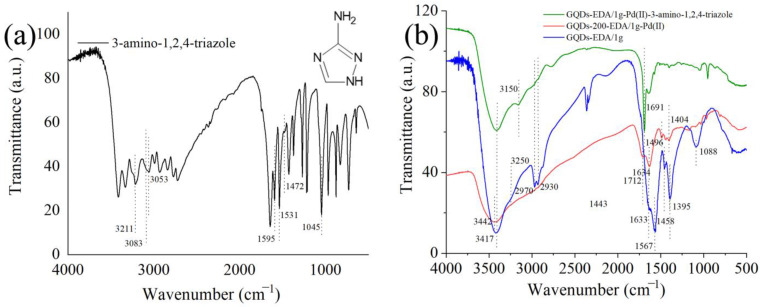
FTIR spectrum and structure of 3-amino-1,2,4-triazole (**a**), and FTIR spectra of GQDs-EDA/1 g, GQDs-EDA/1 g-Pd^2+^, and GQDs-EDA/1 g-Pd^2+^-3-amino-1,2,4-triazole (**b**).

In Figure 15a, an FTIR spectrum of 3-amino-1,2,4-triazole is presented. The bands at 3083 cm^−1^ and 3054 cm^−1^ are assigned to C-H aromatic vibrations [59]. A strong band at 3211 cm^−1^ is attributed to stretching vibrations of N-H bonds in amino groups [59], while those at 1531 and 1472 cm^−1^ originated from C=C stretching vibrations of aromatic domains in 3-amino-1,2,4-triazole molecules [59]. High-intensity bands located at 1595 and 1045 cm^−1^ indicate the presence of endocyclic N=N and C-N-C, respectively [60].

In Figure 15b, FTIR spectra of GQDs-EDA/1 g, GQDs-EDA/1 g-Pd^2+^, and GQDs-EDA/1 g-Pd^2+^-3-amino-1,2,4-triazole are presented. To explore the mechanism of 3-amino-1,2,4-triazole detection using Pd^2+^ ions, firstly FTIR spectrum of GQDs-EDA/1 g in the presence of ions is analyzed. By comparing the spectrum of GQDs and the one recorded in the presence of Pd^2+^ ions, several changes are observed. First, the high-intensity band at 3417 cm^−1^ is slightly reduced and shifted to 3442 cm^−1^ due to complex formation [61]. Bands at 2970, 2930, and 2870 cm^−1^ in irradiated GQDs attributed to stretching vibrations of C-H bonds in -CH and -CH_2_ groups completely disappeared after the addition of Pd^2+^ ions. Band located at 3250 cm^−1^ attributed to stretching vibration of N-H group is also reduced in the presence of Pd^2+^ ions as a consequence of their coordination with amino groups [62]. The band at 1633 is moved to 1712 cm^−1^ and became more pronounced due to the metal–ligand complex formed between the carboxyl group and Pd^2+^ [61]. The band at 1567 assigned to the C=C bond is shifted at 1634 cm^−1^ which confirmed cation-π interactions [63,64]. The band at 1458 cm^−1^ attributed to C-N vibrations of amide groups in GQDs is reduced and moved at 1496 cm^−1^ due to the building of a complex, where N atoms play the role of electron donor [65]. Another change is observed: the band at 1395 associated with C-O is reduced and moved at 1404 cm^−1^ due to surface complexation [66,67]. Additionally, the band at 1088 cm^−1^ attributed to C-OH vibrations completely vanished.

**Scheme 2:**
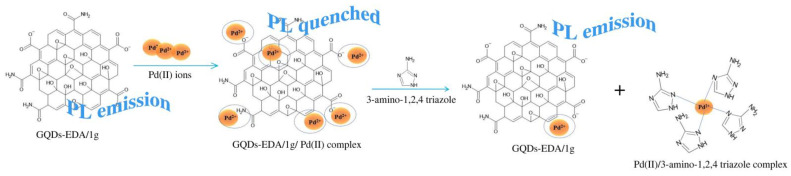
Turn-off/turn-on mechanism of PL detection of 3-amino-1,2,4-triazole using Pd^2+^ as a PL quencher.

After the addition of 3-amino-1,2,4 triazole, the band at 3417 cm^−1^ is fully recovered, due to releasing of -OH groups from the complex with Pd^2+^. The band at 3250 cm^−1^ is also recovered and moved at 3150 cm^−1^. The bands at around 2970 and 2930 cm^−1^ are also partially rehabilitated. The one at 1630 cm^−1^ is completely released from the complex and moved at 1691 cm^−1^. The band assigned to the C=C bond is not fully recovered as well as not returned to the previous position, which proved incomplete regeneration of the π-conjugated system of GQDs. After introducing a 3-amino-1,2,4 triazole in the GQDs-EDA/1 g-Pd^2+^ system, bands at 1458, 1395, and 1088 cm^−1^ are not located. These changes indicate that there is no re-establishment of the C-N and C-O bonds. Results obtained by FTIR analysis confirmed a proposed mechanism of 3-amino-1,2,4 triazole detection whereas PL of GQDs was quenched in the presence of Pd^2+^ ion, after the addition of pesticide was restored (Scheme 2).

### 3.3. Photo-Induced Antibacterial Activity

In order to explore a possible application of amide functionalized GQDs in antibacterial photodynamic therapy, we treated two representative Gram-positive and Gram-negative bacterial lines, *Streptococcus aureus* (*S. aureus*) and *Escherichia coli* (*E. coli*), respectively, with different concentrations of irradiated GQDs and exposed them to blue lights for 16 h. Our previous study showed that p-GQDs induced bacterial cell death at a concentration of 125.00 mg mL^−1^ for *S. aureus*, and 62.5 mg mL^−1^ for *E. coli* [68]. In the same study, it was found that carbon quantum dots with amino functional groups (CQDNH) caused the death of *S. aureus* at 62.5 mg mL^−1^ and *E. coli* at 31.25 mg mL^−1^. In the tested range of concentrations (0.8 to 0.025 mg mL^−1^), GQD-EDA/1 g and GQD-EDA/10 g did not show antimicrobial activity (Table 4).

In parallel, for the sample GQD-EDA/1 g, OD values were obtained to determine whether there was a reduction of OD values in the negative control (Table 5). It was observed that a higher optical density was observed in the treatment compared to the control. Therefore, this method did not show to be reliant on assessing antimicrobial activity. Therefore, the results of antimicrobial activity were read exclusively by resazurin reaction and reinoculation to the appropriate nutrient medium.

Although our previous studies showed the ability of GQDs to induce bacterial cell death when they were exposed to blue light [68], these experiments revealed that GQDs with both amide and amino functional groups lost their phototoxic activity. Our previous studies revealed that GQDs are very potent singlet oxygen produced [8,68,69,70]. Thanks to this ability, together with small size and fast internalization, dots induced oxidative stress in bacterial cells. Here, the introduction of amide and amino groups probably resulted in the inability of dots to transfer the energy toward molecular oxygen. Studies presented in Table 6 showed that amino functionalized and N-doped GQDs are able to induce death of various bacterial strains under conditions presented in the table.

As Table 6 shows, there is a potential for studying these dots under different illumination conditions.

## 4. Conclusions

In summary, we successfully introduced N atoms in GQDs structure in the form of amide groups in a simple, easy, and eco-friendly process using gamma irradiation. At a dose of 200 kGy and 1, 5, and 10 g weight of EDA in the samples, the percentages of introduced nitrogen varied from 6.38–7.16%. FTIR, XPS, and NMR spectroscopies confirmed the presence of N in GQDs structure in the form of amide and amino moieties. After irradiation in the presence of EDA, PL QY was enhanced from 1.45 to 7.24, 6.77, and 5.82% in the GQDs-EDA/1 g, GQDs-EDA/5 g, and GQDs-EDA/10 g, respectively. GQDs-EDA/1 g were investigated as a potential platform for PL detection of carbofuran, and 3-amino-1,2,4-triazole. Emission spectra of GQDs-EDA/1 g recorded in the presence of CF show a linear turn-on response (R^2^ = 0.98) with LOD 5.4 μmol L^−1^. Pesticide 3-amino-1,2,4-triazole was detected through a turn-off/turn-on mechanism using Pd(II) ions as PL quencher. With concentration increase (0–12 μmol L^−1^), PL was resolved linearly (R^2^ = 0.96). LOD was 2.03 μmol L^−1^. This is the first time that PL detection of 3-amino-1,2,4-triazole using GQDs as a potential probe has been reported. Using FTIR spectroscopy showed the formation of hydrogen bonds between GQDs-EDA/1 g and carbofuran. Produced dots were unable to induce an antibacterial response on *E. coli* and *S. aureus*. Although illumination with blue light was applied for 16 h, bacterial cells survived, indicating than modified dots are not phototoxic towards selected bacterial lines under these conditions.

## Figures and Tables

**Table 1:** Atomic % of C, O, and N, and functional groups ratios in the samples p-GQDs, GQD-EDA/1 g, GQDs-EDA/5 g, and GQDs-EDA/10 g.

Sample	%C	%O	%N
p-GQDs [35]	63.00	37.00	0.00
GQDs- EDA/1 g	76.25	17.36	6.39
GQDs- EDA/5 g	77.18	16.10	6.71
GQDs- EDA/10 g	76.60	16.35	7.05

**Table 2:** Data obtained by recording emission spectra of p-GQDs and irradiated N-doped GQDs, at different excitation wavelengths indicated as superscript “Ex”.

Sample	Peak Position (nm)	Intensity/cps
p-GQDs	430^Ex 300^ 437^Ex 320^ 454^Ex 340^ 464^Ex 360^ 472^Ex 380^ 492^Ex 400^	1,068,712 1,504,852 1,595,944 1,522,472 1,217,330 833,934
GQDs-EDA/1 g	441^Ex 300^ 436^Ex 320^ 439^Ex 340^ 447^Ex 360^ 457^Ex 380^ 465^Ex 400^	1,095,082 1,682,314 2,197,876 2,599,566 2,590,304 2,584,144
GQDs-EDA/5 g	438^Ex 300^ 439^Ex 320^ 442^Ex 340^ 446^Ex 360^ 458^Ex 380^ 465^Ex 400^	1,239,596 1,931,034 2,576,008 3,029,036 3,071,398 2,936,478
GQDs-EDA/10 g	439^Ex 300^ 436^Ex 320^ 439^Ex 340^ 444^Ex 360^ 458^Ex 380^ 466^Ex 400^	941,924 1,455,166 1,916,766 2,265,520 2,272,670 2,092,036

**Table 3:** The values of relative QY at the excitation wavelength of 360 nm.

Sample	QY_360_ (%)
p-GQDs [35]	1.45
GQDs-EDA/1 g	7.24
GQDs-EDA/5 g	6.77
GQDs-EDA/10 g	5.82
Rhodamine B	31

**Table 4:** Minimum inhibitory concentrations of GQD-EDA/1 g and GQDs-EDA/10 g (in mg mL^−1^).

Bacterial Strain	GQD-EDA/1 g		GQD-EDA/10 g	
	MIC	MBC	MIC	MBC
*E. coli* ATCC 35218	>0.8	-	>0.8	-
*S. aureus* ATCC 29213	>0.8	-	>0.8	-

**Table 5:** OD values for bacterial strains *E. coli* ATCC 35218 and *S. aureus* ATCC 29213 treated with GQD-EDA/1 g.

OD Values			
*E. coli*		*S. aureus*	
0.85	0.77	0.63	0.49
0.78	0.70	0.51	0.50
0.67	0.61	0.50	0.43
0.69	0.63	0.90	0.42
0.66	0.62	0.47	0.50
0.9	0.89	0.50	0.47
0.91	0.72	0.52	0.56
0.05	0.05	0.05	0.05

**Table 6:** Studies regarding GQDs with N in structure and its antibacterial photodynamic efficiency.

Material	Bacterial Strain	Illumination Wavelength (WL)	Effects
nitrogen-doped GQDs and amino-functionalized N-GQDs (amino-N-GQDs) [71]	*Bacillus subtilis*	WL 720–820 nm, power of 2.8 mW, 394.24 nJ pixel^−1^, exposure time 3.171 s	amino-N-GQDs6 μg mL^−1^ 100% elimination
amino-N-GQDs [72]	Methicillin-resistant *Staphyococcus* *aureus* (MRSA)	power: 239.36 nJ pixel^−1^, exposure time 12 s, WL 800 nm	0.25 µg mL^−1^ Incubation 3 h at 37 °C in the dark
octa-imine substituted Zinc phthalocyanines with nitrogen, sulfur co-doped graphene quantum dots (N,S-GQDs) [73]	*S. aureus*	a diode laser WL 670 nm, 20 MHz repetition rate, 44 ps pulse width	80 min irradiation 100% elimination
amino-N-GQDs [74]	*E. coli*	WL 960 nm, laser source, 222.7 nJ pixel^−1^, total illumination: ~1.11 s	0.75 µg mL^−1^ elimination > 99%

## Data Availability

Not applicable.
